# Stabilization of Curcumin by Complexation with Divalent Cations in Glycerol/Water System

**DOI:** 10.1155/2010/292760

**Published:** 2010-06-16

**Authors:** Bachar Zebib, Zéphirin Mouloungui, Virginie Noirot

**Affiliations:** ^1^Université de Toulouse-UMR 1010, Laboratoire de Chimie Agro-Industrielle, ENSIACET, INPT, INRA, 4 allée Emile Monso, 31432 Toulouse Cedex 4, France; ^2^Laboratoires Phodé S.A.S., ZI Albipole 81150 Terssac, France

## Abstract

The purpose of present study was to stabilize curcumin food pigment by its complexation with divalent ions like (Zn^2+^, Cu^2+^, Mg^2+^, Se^2+^), in “green media” and evaluate its stability in vitro compared to curcumin alone. The curcumin complexes were prepared by mechanical mixture of curcumin and sulfate salts of each metal (metal : curcumin 1/1mol) into unconventional and nontoxic glycerol/water solvent. Two stoichiometry of complex were obtained, 1 : 1 and 1 : 2 (metal/curcumin), respectively. On evaluation of in vitro stability, all complexes were found to provide a higher stability from curcumin alone.

## 1. Introduction

**Figure 1:**
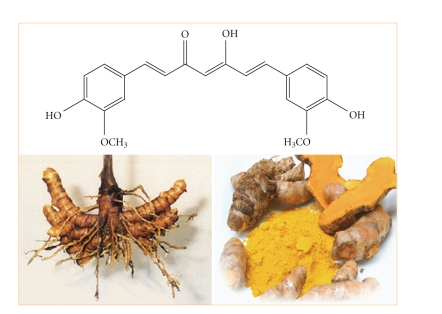
Photo of rhizomes of Curcuma longa Linn plant and chemical structure of polyphenolic curcumin compound.

Naturalpigment is a vital quality attribute of foods, and plays an important role in sensory and consumer acceptance of products [1–3]. Curcumin, a naturally occurring polyphenolic phytoconstituent, isolated from the rhizomes (Figure 1) of *Curcuma longa Linn*, is an important permitted natural colorant used in food, nutritious, and pharmaceutical preparations among others [4–8]. Curcumin is chemically (1*E*,6*E*)-1,7-bis(4-hydroxy-3-methoxyphenyl)hepta-1,6-dienne-3,5-dione. It has a pKa_1_, pKa_2,_ and pKa_3_ value of  7.8, 8.5, and 9.0, respectively, for three acidic protons [9]. It is insoluble in water under acidic or neutral conditions but dissolves in alkaline conditions. Curcumin is unstable undergoing rapid hydrolytic degradation in neutral or alkaline conditions to feruloyl methane and ferulic acid [10]. Since it is insoluble in aqueous medium and has poor stability towards oxidation, light, alkalinity, enzymes and heat, it cannot really be widely used in food and pharmaceutical processing industry [11, 12]. Although, exposure of the curcumin pigment to alkaline foods or ingredients may be difficult to avoid. For these reasons, it should be protected curcumin in certain forms from physical and chemical damage before its industrial application. 

 Complexation of curcumin with transition metals has attracted much interest over the past years as one of the useful requirements for the treatment of Alzheimer's disease [13, 14] and in vitro antioxidant activity [15].

 Moreover, several metallocomplexes of curcumin have been synthesized characterized and evaluated for various biological activities [15–19]. However, all these metallocomplexes of curcumin have been prepared under relatively high temperature synthesis conditions (reflux at 100°C under nitrogen gas for 3 hours) in the presence of organic solvent like ethanol, methanol, or acetone. Other recent studies [20, 21] suggest the preparation of curcumin-phospholipid complex under smooth synthesis conditions (temperature not exceeding 60°C for 2 hours) for hepatoprotective application. However, organic solvents like dichloromethane and hexane are not excluded from experimental preparation of curcumin-phospholipid complex. 

 One of the interests of our group is to develop new “green routes” to protect and stabilize natural biomolecules using glycerol chemistry for food, pharmaceutical, agriculture, and cosmetic applications. In this paper, we investigate a complexation of curcumin with divalent ions such (Zn^2+^, Cu^2+^, Mg^2+^, Se^2+^) by mechanical mixture, without any conventional organic solvents. The preparation of metallocomplexes was carried out in presence of water/glycerol solvent at 25°C. Obtained complex was characterized by IR and UV spectroscopic methods. Supportive evidence of obtained stoichiometry has been suggested from TG-DTA thermal analysis.

## 2. Experimental Section

### 2.1. Materials

Curcumin (95%), glycerol (99%), zinc sulfate (ZnSO_4_·7H_2_O; 22%), Copper sulfate (CuSO_4_·5H_2_O; 25%), Magnesium sulfate (MgSO_4_·7H_2_O; 49.1%), Sodium selenite (Na_2_SeO_3_; 45%), were procured from Phodé Laboratory S.A.S. (Albi, France).

### 2.2. Preparation of M^2+^-Curcumin Complex

Zinc sulfate (ZnSO_4_·7H_2_O; 22%) was mechanically mixed in mortar with curcumin (M^2+^: Curcumin 1/1 mol) until homogenous powder mixture was obtained. Then glycerol/water (1 : 1 v/v) solution was added to mixture followed by mechanical shaking at 25°C until pasty combination was obtained. Then, pasty product was dried in room temperature at 50°C until water evaporation. Free glycerol was eliminated by washing with distilled water. Powder complex of Zn^2+^-curcumin was obtained. Other complexes derived from another ion sulfate source were prepared by the same method.

### 2.3. Assay

The contents of curcumin in complex were determined spectrophotometrically. 5 mg of the complex was dissolved in 10 mL of DMSO (Dimethylsulfoxide) and stirred for 1 hour on a magnetic stirrer. The concentration of curcumin in complex was determined by measuring the absorbance of the solution so obtained at 435 nm.

### 2.4. FT-IR Spectroscopy

FT-IR spectroscopy of curcumin and Zn-curcumin complex was performed on fourrier-transformed infrared spectrophotometer (Bruker VECTOR 22) equipped with a detector DTGS witch resolution is fixed to 4 cm^−1^. The pellets of sample (10 mg) and potassium bromide (200 mg) were prepared by compressing the powders at 5 bars for 5 minutes on KBr press and the spectra were scanned on the wave number range of 4000–850 cm^−1^.

### 2.5. UV-Visible Spectroscopy

HP 8452 diode array UV/visible spectrophotometer was used to record spectra of curcumin and curcumin complex in DMSO solvent. The spectra were scanned on the wave number range of 350–600 nm.

### 2.6. Thermogravimetric Analysis

Thermograms of curcumin complexes were recorded using differential scanning calorimeter (Seiko SSC 5200H). The samples (20–40 mg) were sealed in the platinum crimp pan, and heated at the speed of 10°C /min from 25°C to 600°C in air atmosphere.

### 2.7. In Vitro Stability

In vitro kinetic degradation of curcumin from all curcumin complexes was followed spectrophotometrically between 350 and 600 nm. 10 mg of curcumin complex was incubated at 37°C in 100 mL of buffer solutions at various pH from 2.0 to 10.0. Kinetic degradation reaction of curcumin and its complexes was followed between 0–120 minutes and 0–24 hours, respectively.

## 3. Results and Discussion

The method of preparation of curcumin complexes was found to be reproducible yielding 98% of product. On assaying the complex, it was found to contain about 45%, 60%, 61%, and 50% of curcumin for Mg-curcumin, Cu-curcumin, Zn-crucumin, and Se-curcumin complexes, respectively. 

### 3.1. FT-IR Characterization

**Figure 2:**
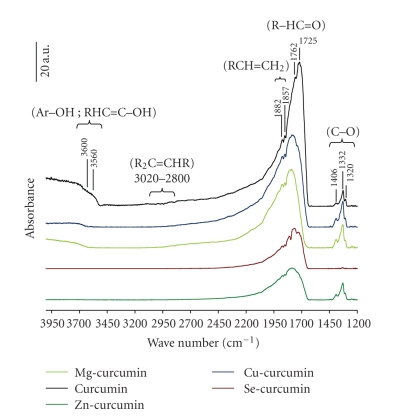
Normalized FT-IR spectra of curcumin complexes compared with that of curcumin alone.

Figure 2 compares the IR spectra of curcumin and all curcumin complexes. The spectrum of curcumin may be assigned as follows:

- Two broad band's at 3600 and 3560 cm^−1^ attributed to vibrations of free hydroxyl-group of phenol (Ar−OH) and alcohol group (R−OH), respectively,
- Two bands at 1882 and 1857 cm^−1^ attributed to vibrations of C−H bond of alkenes groups (RCH=CH_2_),
- An intense band at 1725 cm^−1^ attributed to the vibration of the carbonyl bond (C=O) accompanied by a small shoulder at 1762 cm^−1^ due to Keto-enol tautomerism of curcumin compound,
- Tree bands at 1406, 1332, 1320 cm^−1^ attributed to vibrational mode of C−O elongation of alcohol and phenol groups.

Compared with the reference spectrum of curcumin, all complexes shows a great decrease in the intensity of (C=O) carbonyl band, accompanied by a shift (Δ*ν* = 53–75 cm^−1^) to high wave values (Table 1). In addition, a net decrease in the intensity of the free (OH) hydroxyl group of curcumin was observed in the case of Mg-curcumin and Cu-curcumin complexes, but totally disappeared for Se-curcumin and Zn-curcumin complexes. These two above phenomena indicating that some interaction has occurred at these sites and its involvement in complexation by new created link between metal and curcumin compound. 

### 3.2. UV-Visible Characterization

Curcumin is soluble in most of the organic solvents, lipids, and micellar solutions, and is insoluble in neutral solutions. The curcumin complexe is insoluble in organic solvents like methanol and acetonitrile, and is soluble only in DMSO, and neutral and negatively charged micellar solutions. It is also soluble in lipids and membranes. 

**Figure 3:**
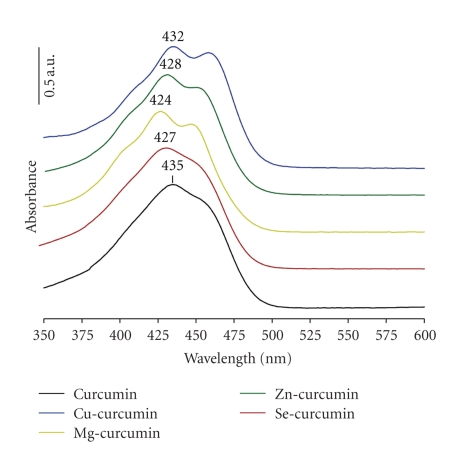
Normalized absorption spectrum of curcumin complexes compared with that of curcumin in DMSO solvent.

 A UV-visible spectrum of the complexes in DMSO showed absorption maximum at 435 nm assigned to the band *π* → π* of curcumin (Figure 3). Compared with curcumin, the complexes in DMSO shows a maximum absorption shifted by (1–8 nm), which varies between (427–434 nm), and the shoulders at (410–413 nm) and (448–451 nm) are attributed to a curcumin → metal (M^2+^) charge transfer, specific complex formed. We believe that the variation of the absorption peak of curcumin and shoulders apparition in different complexes depend on the nature of metal (M^2+^) ion implication.

### 3.3. Thermogravimetric Analysis

**Figure 4:**
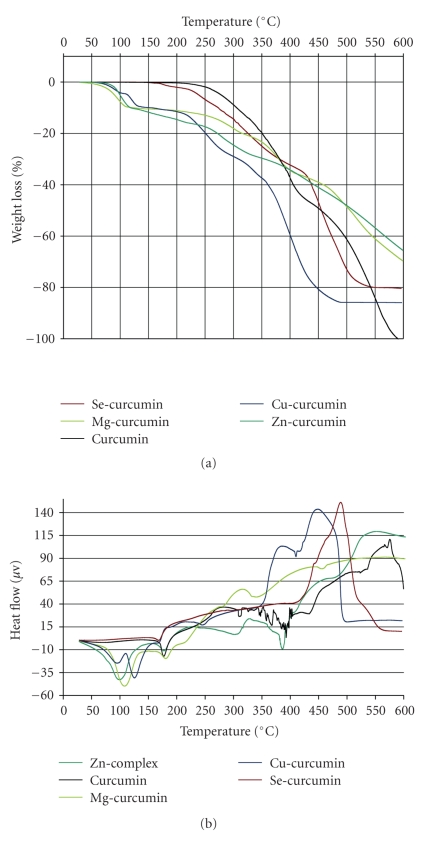
TG and ATG analysis of curcumin and curcumin complexes. Conditions: heating speed 10°C/min. under air atmosphere.

Supporting evidence of the structure of complexes is suggested by thermal analysis. The TG-DTA measurements of all the complexes were performed in air over the temperature range of 25–600°C (Figure 4).

#### 3.3.1. Curcumin

It was thermally stable up to 160°C. Above this temperature we observe an endothermic peak at 174°C (weight loss: found 3.3%, calcd. 3.1%) related to the deshydroxylation of OH groups by elimination of two water molecules. After 400°C curcumin was totally decomposed.

#### 3.3.2. Zn-Curcumin and Mg-Curcumin Complex

They were thermally stable up to 65°C. Above this temperature one molecule of crystalline water is eliminated in one step at 93°C and 102°C, respectively, (weight loss: found 3.2%, calcd. 3.0%). The existence of an anhydrous complexes [Mg(L)(H_2_O)] and [Zn(L)(H_2_O)] can be evident from the plateau between 90 and 150°C. Then, the sample weight of two complexes decreases up to 205°C which is probably connected with the elimination of the coordinated water molecule, leading to the formation of [Mg(L)] and [Zn(L)] species (weight loss: found 11.2%, calcd. 11.4%). The intermediate is stable within the interval of 160–220°C. After 250°C we observe a chemical decomposition of curcumin without formation of thermallystable intermediates up to 600°C. At this temperature ZnO and MgO oxides can be formed.

#### 3.3.3. Cu-Curcumin Complex

It was thermally stable up to 60°C. Above this temperature one molecule of crystalline water is eliminated in two steps at 88°C and 122°C (weight loss: found 2.6% and 7.4%, calcd. 3.0% and 7.2%, resp.). The existence of an anhydrous complexe [Cu(L)(H_2_O)] can be evident. Then, the sample weight of complexe decreases up to 185°C which is probably connected with the elimination of the coordinated water molecule, leading to the formation of [Cu(L)] specie (weight loss: found 11.0%, calcd. 11.2%). At 237°C we observe a small endothermic peak which can be related to the elimination of coordinated Ligand. Chemical decomposition of curcumin between 350–500°C was obtained. A thermally stable decomposition product exists after 500°C. It may be related with the formation of CuO oxide.

#### 3.3.4. Se-Curcumin Complex

It was thermally stable up to 150°C. Onemolecule of crystalline water is eliminated in range of 80–150°C. In the range of 152–190°C (weight loss: found 1.78%, calcd. 1.80%) we observe an endothermic peak centered at 165°C which can be related to the deshydroxylation of OH groups of two curcumin ligands connected to one selenium atom by the elimination of four water molecules (deduced by obtained weight loss). Next decomposition at 487°C is exothermic which can be related to the degradation of total curcumin ligand compounds. Selenium oxide (SeO) can be formed after 550°C. 

**Figure 5:**
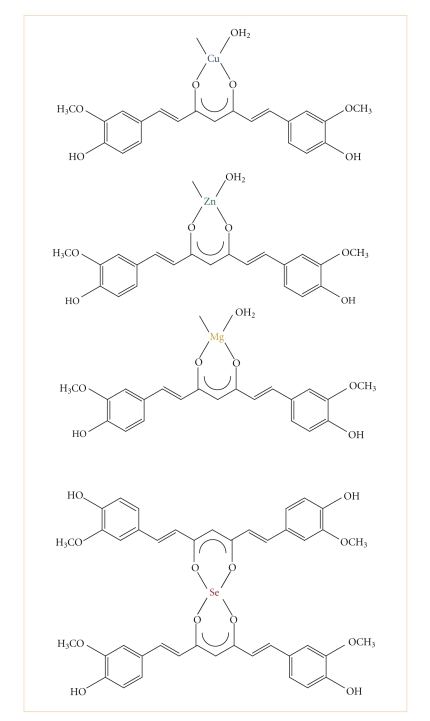
Proposed structures of curcumin complexes based on experimental calculation from TG and DTA measurements.

 Thermograms of curcumin complexes were showed in Figure 4. Thermal analysis proves that two stoichiometry of complexes were obtained (Figure 5), 1 : 1 and 1 : 2 (metal/ion), respectively. This difference of coordination geometry is probably due to the various physicochemical properties of each metal ion involved in complexation reaction, like electro-negativity, nuclear ray, polarity, solubility, and electronic configuration. However, previous study [16, 17] suggest that other factors such, molar ratio of reactants, the nature of solvent used, or the chemical source of metal ion, are implicated in the coordination geometry of resulting complex.

### 3.4. Stability Evaluation

#### 3.4.1. Curcumin

It has been shown that curcumin has a poor light stability. About a 5% decrease in absorbance due to curcumin has been measured during the time for typical sample preparation when clear rather than amber glassware is used [22]. Curcumin decomposes when exposed to sunlight, both in ethanolic and methanolic extracts and as a solid, vanillin, vanillic acid, ferulic aldehyde and ferulic acid have been identified as the degradation products [23]. 

**Figure 6:**
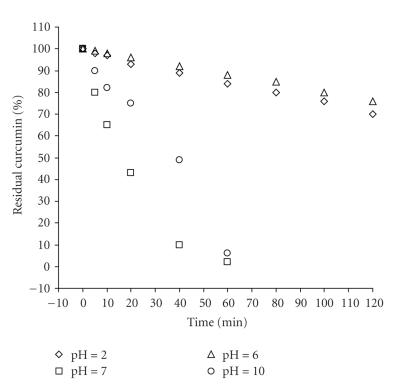
Kinetic degradation of curcumin in various pH of 0.1 M buffer at 37°C. The data are normalized to a value of 100 at zero time.

 When curcumin was added to 0.1 M phosphate buffer, pH = 7 (physiological condition in vitro), the majority of curcumin was degraded after 1 hour. A series of pH values from 2 to 10 in buffer solutions were assayed for this degradation. Figure 6 shows the kinetics of curcumin degraded at various pH values, 37°C, using HP 8452 diode UV/visible spectrophotometer. Entities percent of the residual curcumin concentration versus time were reasonably linear at all pH values tested, indicated that degradation followed apparent first-order kinetics at 37°C. 

 In present work, we found that more than 90% of curcumin decomposed rapidly in buffer systems at neutral-basic pH conditions. The increased stability of curcumin in acidic pH condition may be contributed by the conjugated diene structure. However, when the pH is adjusted to neutral-basic conditions, proton removed from the phenolic group, leading to the destruction of this structure.

#### 3.4.2. Curcumin Complex

The kinetics of demetallation of curcumin complex was carried out in various pH buffers. The complexes decompose via general reaction

(1) $$\begin{array}{cc} & x{\text{ML}}_{n(\text{total})}+\text{Buffer}\;\text{solution}\\ & \;\to \left(x-1\right){\text{M}}^{2+}+n\left(x-1\right){\text{L}}_{\left(\text{degraded}\right)}+y{\text{ML}}_{n(\text{residual})}, \end{array}$$

where degraded curcumin (L_(degraded)_) entity is not measured spectrophotometrically. 

 In acidic media, complexes are decomposed via

(2) $${\text{ML}}_{\left(\text{total}\right)}+2{\text{H}}^{+}\to {\text{M}}^{2+}+{\text{H}}_{2}{\text{L}}_{\left(\text{degraded}\right)},$$

where M = Metal ion and L = Ligand = curcumin.

**Figure 7:**
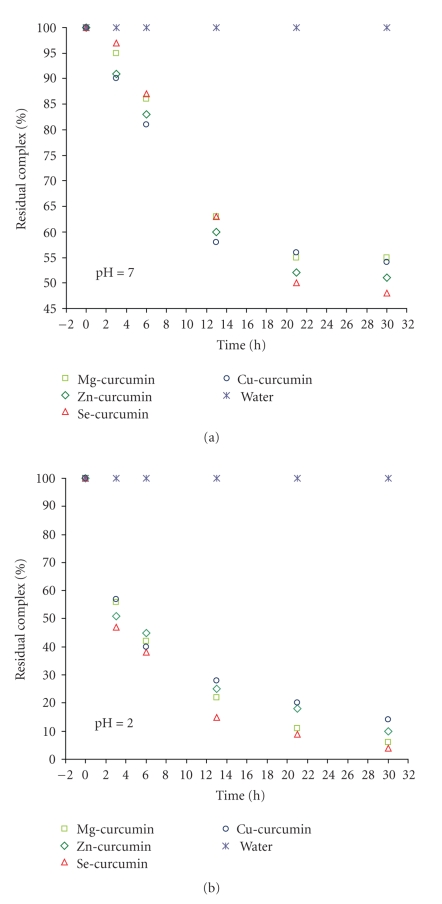
Kinetic degradation of curcumin complexes in water (pH = 6.5) and at pH 2 and 7 of 0.1 M buffer at 37°C. The data are normalized to a value of 100 at zero time.

 It was found that all complexes were very stable in purified water (pH = 6.5) up to 30 hours at 37°C. All complexes rapidly decomposed at acidic pH 2 (Figure 7) and the dissociation of complexes was decreased in higher (basic) pH 10.0 (data not shown), indicating that the stability of complexes was dependent on proton concentration. As presented in Figure 7, the dissociation of complexes was in equilibrium by 50% degradation in buffer pH 7.0. The dissociation was found to be 90% in acidic buffer (pH 2). All decomposed complexes reaching an equilibrium constant level after 13 hours in buffer pH 2.0 and 7.0.

**Figure 8:**
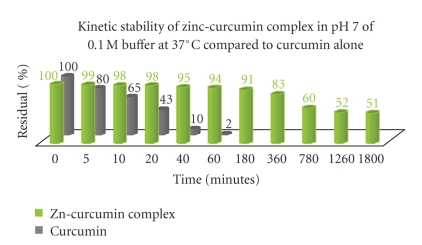
Kinetic stability of zinc-curcumin complex compared to curcumin alone in pH 7.0 of 0.1M buffer at 37°C.

However, stability of these complexes compared to curcumin alone, for the same time interval, is much higher. As an indication, at buffer pH 7.0, curcumin was totally degraded after 1 hour, while in the same conditions; less than 5% of complex was degraded (Figure 8). Therefore, the stability of curcumin at pH 7 has been multiplied by a factor of 20 after its complexation with metal ions.

## 4. Conclusion

For the first time, curcumin complexes with divalent ions were successfully prepared in “green media” glycerol/water solvent. Complexes were characterized by spectroscopic (IR, UV) and thermogravimetric analysis. Their physicochemical stability was evaluated “in vitro,” where conditions are close to those physiological. In FT-IR, metal-curcumin link was highlighted by the decrease of carbonyl band (CO) intensity of curcumin coupled with a shift (70 nm) to high wave numbers. The linkage was also confirmed by UV analysis where charge transfer between curcumin and metal (curcumin→ M^2+^) is observed. Supporting structure of synthesized complexes was suggested by thermal analysis which shows two different stoichiometry (1 : 1 and 1 : 2 (ion/curcumin)). Indeed, we prejudge each structure has different physicochemical properties such a conductivity, polarity, electro-negativity, molecular size, and carrying and release of active compound (curcumin), which is not without effect on the biological applications. 

 Furthermore, the created connection between molecules is based on electrostatic links between curcumin and oligoelements, which is reversible on the action field, for example, in rural tract. These physical links have shown from “in vitro” studies that they are able to protect curcumin against chemical degradation in neutral and basic media for long period of time (30 hours), which gives much hope for its industrial application. 

 On the other hand, we believe that curcumin complex synthesis in an unconventional and nontoxic solvent, for example, glycerol and its derivatives (glycerol fatty esters) opened a new path for stabilization of active ingredients in food and pharmaceutical field were complex can be formed “in situ” inside the product formulation health without the requirement of solvent isolation. Beside, glycerol and its derivatives can play a good role in the solubilization and diffusion of many organic molecules insoluble or poorly soluble in water without forgotting its other conventional roles that comes in many applications.

## Figures and Tables

**Table 1:** Wave length changes of main vibration modes from infrared (KBr pellets) spectral data of curcumin and curcumin complexes. Vibrational modes: (*ν*) stretching; (*δ*) in-plane bending; (—) not observed.

Assignment	*ν*_IR_ (cm^−1^)				
	Curcumin	Mg-curcumin	Cu-curcumin	Zn-curcumin	Se-curcumin
*ν* ^enol^_(O–H)_	3560	—	—	—	—
*ν* ^Phenol^_(O–H)_	3600	—	—	—	—
*ν* ^Alkene^_(C–H)_	1882	1885	1885	1891	1890
	1857	1865	1860	1868	1865
*ν* ^Ketone^_(C=O)_	1762	—	—	—	1738 (Δ*ν* = 13)
	1725	1800 (Δ*ν* = 75)	1795 (Δ*ν* = 70)	1800 (Δ*ν* = 75)	1778 (Δ*ν* = 53)
*δ* ^enol^_(C–O)_	1406	1402	1402	—	1406
*δ* ^Phénol^_(C–O)_	1332	1337	1337	—	1337
	1320	1315	1315	—	1315
